# Accuracy of modified CAD/CAM generated wafer for orthognathic surgery

**DOI:** 10.1371/journal.pone.0216945

**Published:** 2019-05-16

**Authors:** Jin Hoo Park, Yong-Bin Lee, Sang Yoon Kim, Hyung Jun Kim, Young-Soo Jung, Hwi-Dong Jung

**Affiliations:** 1 Department of Oral & Maxillofacial Surgery, Oral Science Research Institute, Yonsei University College of Dentistry, Seoul, Korea; 2 Department of Oral & Maxillofacial Surgery, Hankook General Hospital, Cheongju, Korea; 3 Attending, INOVA Fairfax Hospital, Falls Church, VA, USA, Former Chief Resident at Harvard/MGH Oral and Maxillofacial Surgery, Boston, MA, United States of America; Navodaya Dental College and Hospital, INDIA

## Abstract

The aim of this study was to investigate an accuracy of modified CAD/CAM generated wafers for orthognathic surgery. A total of 20 patients who had undergone bimaxillary orthognathic surgery were included and divided into two groups: A conventional CAD/CAM generated intermediate wafer and a modified CAD/CAM generated intermediate wafer. A series of CT images were taken to compare the virtual simulations with the actual postoperative outcomes(1 month after surgery). In conventional group, the mean difference of maxillary position between virtual simulation models and postoperative results was 0.78mm and overall average error within 1mm was observed in 66.4% of the repositioned maxilla. In modified group, the mean difference was 0.77mm and overall average error within 1mm was observed in 68.3%. There were no significant statistic differences between two groups in maxillary position. This study suggests that the CAD/CAM generated wafer provides excellent accuracy. The modified CAD/CAM wafer was only comparable to conventional design in accuracy and it cannot guarantee the superior precision. However, the modified design could be beneficial in cases with unstable condylar position or for inexperienced surgeons.

## Introduction

Diagnosing and treatment planning for corrective jaw surgery based on 2-dimensional(2D) cephalometric radiographs have been successful for a few decades.[1] However,there are many sources for errors during the preoperative treatment planning phases and intraoperative repositioning of bony segments to a planned position in cases of severe dentofacial deformity requiring complex maxillomandibular movement.

The importance of 3D virtual diagnosis and planning was highlighted to overcome the limitations of the traditional method.[2–4] By virtue of computed tomography(CT) imaging and 3D printing technique, 3D virtual planning with fully digitized patients’ data have been studied and the surgical wafer can be fabricated from these data.[3,5,6] Furthermore, computer-aided design (CAD) and computer-aided manufacturing(CAM) technique for surgical splints was introduced in order to reduce the errors associated with traditional laboratory procedures. Some studies have been carried out to evaluate the accuracy of CAD/CAM generated wafers and it has been provento be clinically acceptable.[7–9] For the higher degree of precision, customized surgical guides have been developed with or without conventional occlusal based wafer.[10,11]

The aim of this study is to evaluate and to compare the accuracy of two types of CAD/CAM generated wafers in orthognathic surgery by comparing virtually planned versus actual postoperative 3D reconstructed models.

## Materials and methods

This study followed the Declaration of Helsinki regarding medical protocol and ethics, and was approved by the Institutional Review Board of Yonsei Dental Hospital(IRB No.2-2016-0007).

**Fig 1:**
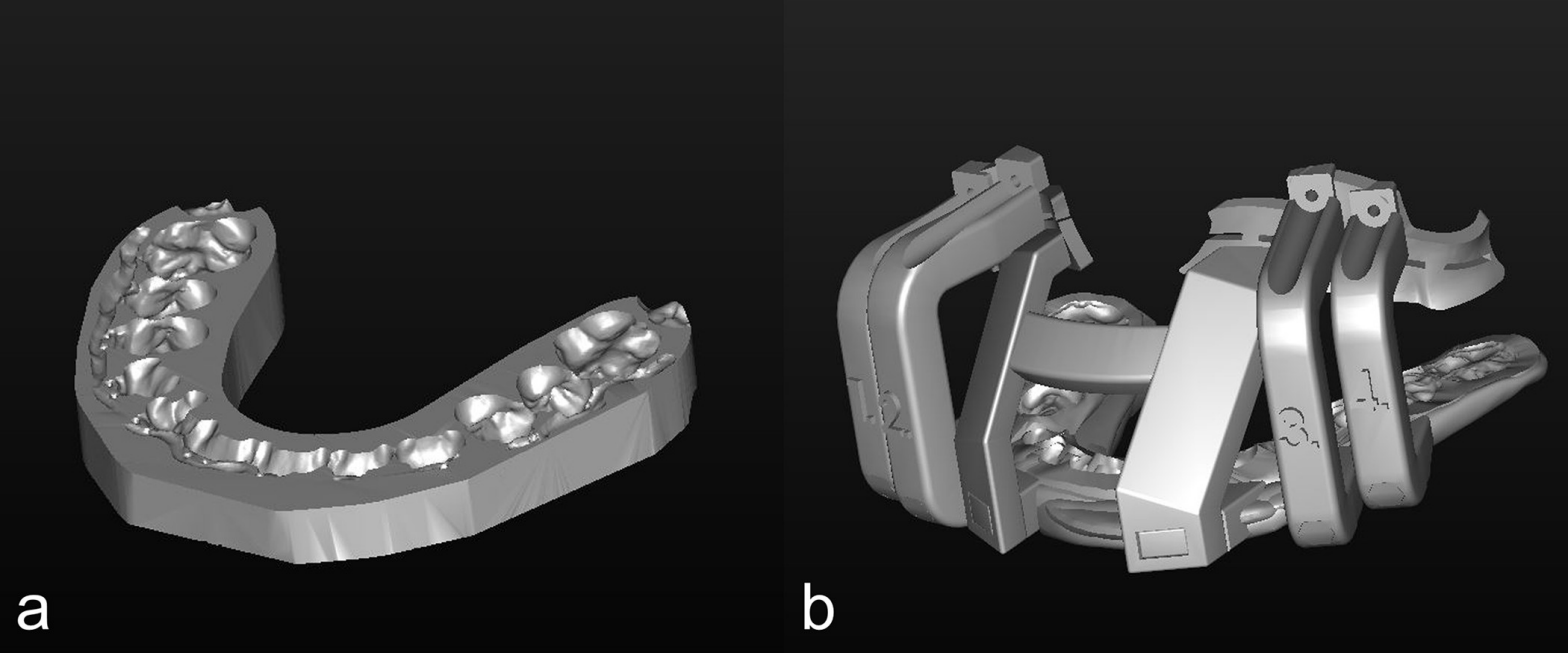
Conventional wafer and modified wafer. a. Conventional wafer. b. Modified wafer.

This retrospective study investigated consecutive20 adult patients who underwent bimaxillary orthognathic surgery by a single surgeon between December 2014 and March 2016 at the Department of Oral and Maxillofacial Surgery. All patients were diagnosed with mandibular prognathism with or without facial asymmetry, and were treated with conventional Le Fort I osteotomy and intraoral vertical ramus osteotomy. All the procedure was completed with maxillary surgery first approach, and the final wafer was applied for mandibular setback. Patients were divided into two groups according to the type of CAD/CAM surgical wafers used: conventional group(n = 10), conventional occlusal based wafer; modified group(n = 10), modified surgical guide.(Fig 1) There was no difference in severity of deformity between two groups. The patients were examined with cone beam computed tomography(CBCT) (Alphard 3030, Asahi Roentgen Inc, Kyoto, Japan) in conventional group and multislice CT(MSCT) (GE Medical System, Milwaukee, U.S.A) in modified group preoperatively(T0) and 1 month postoperatively(T1). Since modified CAD / CAM wafers must be in intimate contact with the maxillary bone surface during surgery, MSCT was taken in the modified group to obtain a clearer bone surface.

All acquired (CB)CT data were stored in Digital Imaging Communication in Medicine(DICOM) format and reconstructed into 3D images using Mimics 16.0 software program(Materialisen.v., Leuven, Belgium). To eliminate significant artifacts from orthodontic appliances and dental restorations, laser scanning of dental plaster models was done and stored in Standard Tessellation Language(STL) file format. The 3D virtual skull models were constructed using these data. Simplant Pro 14.0 software program(Materialise Dental n.v., Leuven, Belgium) was used for preoperative 3D diagnosis, planning, and virtual simulation surgery. The three reference planes were set up to achieve ideal repositioning of maxilla and mandible as followed.

- Frankfort Horizontal Plane(FHP): the plane defined by midpoint of bilateral Porion, right and left Orbitale
- MidSagittal Plane(MSP): the plane through Nasion and center of foramen magnum and perpendicular to the FHP
- N-coronal plane: the plane through Nasion and perpendicular to the plane FHP & MSP

**Fig 2:**
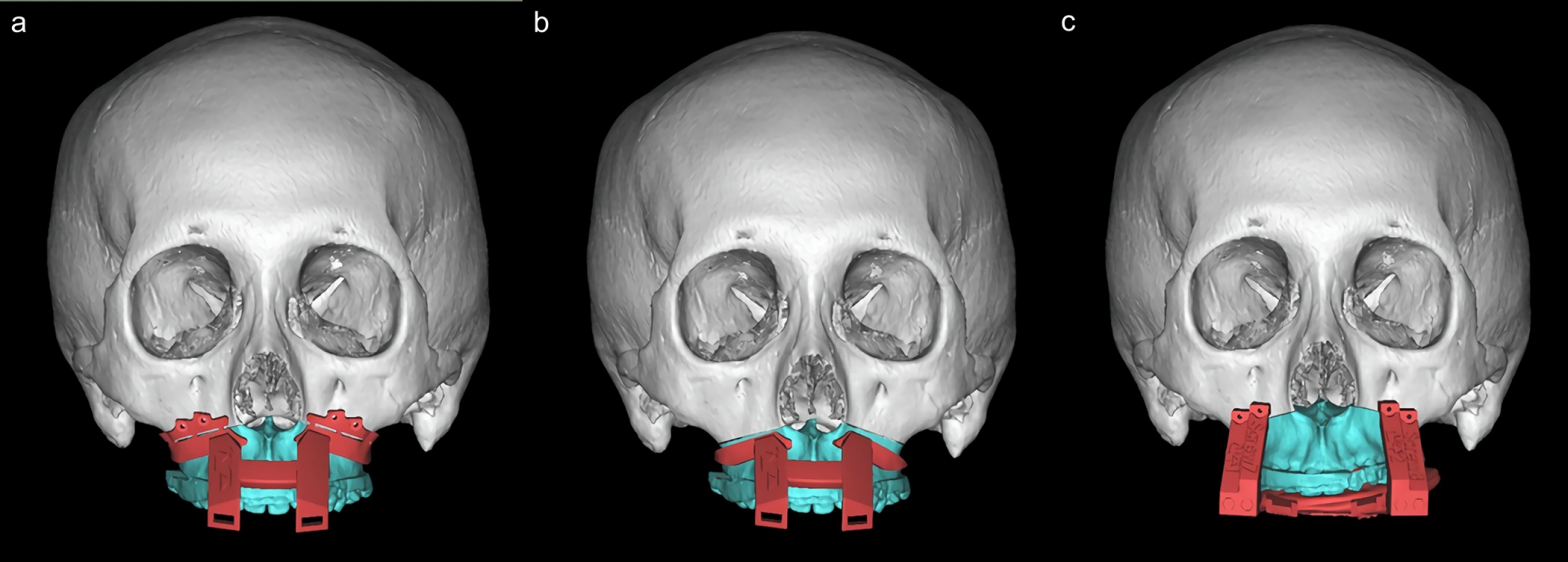
Fabrication of modified CAD/CAM generated wafer. a. Osteotomy guide. b. Resection guide. c. Repositioning guide.

Virtual osteotomy and movement of bony segments were performed and the position of mandible was determined according to the final occlusion which was reviewed and discussed with an orthodontist. The amounts of movement of bony segments were controlled and quantified. Virtual simulated 3D models were saved into STL file format and surgical wafers were fabricated using CAD/CAM technique for both groups. Traditional occlusal-based intermediate wafer was fabricated for conventional group and the modified wafer which consisted of osteotomy guide(Fig 2A), resection guide(Fig 2B), and repositioning guide(Fig 2C) was fabricated for modified group. This modified surgical guide with occlusal -based wafer can be adapted and fixated to the anterior maxillary walls with screws.

In conventional group, Le Fort I osteotomy wasper formed by the conventional method. Intermaxillary wiring was performed with the conventional occlusal-based intermediate wafer. Maxilla was repositioned while confirming maxilla bone interference through mandible hinge movement. The vertical position of maxilla was measured using a K-wire inserted into the glabella. In modified group, an osteotomy guide was fixated with 4 screws and it allowed precise location of the initial osteotomy line. Two screw holes made for osteotomy guide were used for a precise placement of repositioning guide which allows accurate osteotomy and internal fixation as planned. A resection guide was applied following Le Fort I osteotomy, which indicated the amount of bony interferences for maxillary repositioning. Resection guide was used for reduction of bony interference quickly and precisely. Then, a repositioning guide was fixed to the 4 pre-drilled screw holes with same screws, which guides the maxilla to the planned position. Maxillary position was confirmed with the use of repositioning guide. The use of repositioning guide can potentially eliminate the need for external landmark measurement methods. Seven of all subjects were treated with additional genioplasty and the other seven were treated with additional chin shaving. Postoperative intermaxillary fixation(IMF) was applied for 10 days followed by continued active physical therapy and IMF using elastics.[12]The final wafer was fixed on upper dentition and maintained until the end of physical therapy.

**Fig 3:**
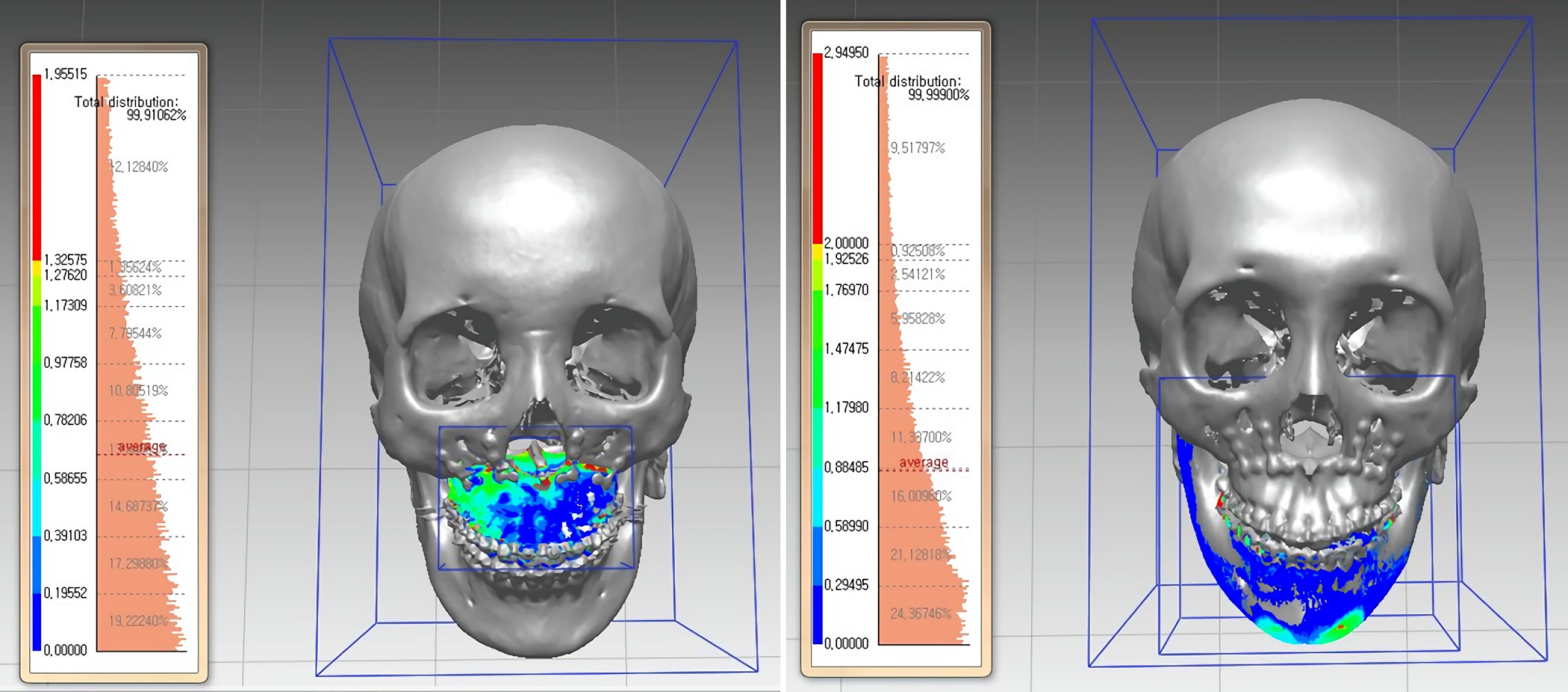
Calculation of surface discrepancy between virtual planning model and 1-month postoperative result. a. Surface discrepancy of Maxilla. b. Surface discrepancy of Mandible.

To evaluate the accuracy of the wafers, the virtual simulated and actual postoperative 3D models were compared. Superimposition using Mimics (Materialise`s Interactive Medical Image Control System, Belgium) and Rapidform2006 software(INUS Technology, Seoul, Korea) of two 3D models was performed and the surface discrepancies(errors) between the virtual and actual positions of maxilla and mandible were measured. The cranium was not influenced by orthognathic surgery, thus this region was used as fiducial area. For the patients with additional genioplasty or chin shaving, the errors were measured except chin area.(Fig 3)

The Mann-Whitney test was performed to confirm the significance of the surface discrepancies between virtual simulated position and actual postoperative position of bony segments. Statistics were considered significant at P< 0.05. Statistical analyses were performed with the SPSS version 18.0 software (SPSS Inc. Chicago, Il, U.S.A).

## Results

A total of 20 patients who underwent bimaxillary surgery were enrolled and divided into two groups: a conventional group (n = 10) and a modified group (n = 10). Patients in conventional group were aged between 17 to 22 years old with a mean age of 19 and 4 were males. Patients in modified group were aged between 17 to 32 years old with a mean age of 21.2 and 2 were males.

In maxilla, the mean surface discrepancy was 0.78 ± 0.13mm and error within 1.0mm was shown 53.9~88.4% (mean 66.4%) of the bony surface in conventional group. In modified group, the mean surface discrepancy was 0.77 ± 0.08mm and error within 1.0mm was shown 52.2%~75.8% (Mean 68.3%). There were no significant statistical differences between both groups. Furthermore, most cases showed the errors within 2mm except one case per group.(Table 1.)

**Table 1:** Discrepancy between virtual plan and postoperative result at maxilla.

	Conventional Group	Modified Group	*p*-value
Average distance(mm)	0.78 ± 0.13	0.77 ± 0.08	0.393
Average error within 1mm(%)	66.4	68.3	0.19

In mandible, the mean surface discrepancy was 0.93 ± 0.35mm in conventional group and 1.21 ± 0.24mm in modified group. Although the modified group presented greater discrepancy, there were no statistically significant differences. The surface area error less than 1.0mm was 35.4~88.5% (mean 61.8%) and 33.2~66.3%(mean 47%), respectively. The difference between two groups was not statistically significant. (Table 2.)

**Table 2:** Discrepancy between virtual plan and postoperative result at mandible.

	Conventional Group	Modified Group	*p*-value
Average distance(mm)	0.93 ± 0.35	1.21 ± 0.24	0.075
Average error within 1mm(%)	61.8	47.0	0.089

## Discussion

The intermediate surgical wafer is one of the most important factors in orthognathic surgery to achieve an accurate position of maxilla. However, it had been reported that the traditional intermediate surgical wafer could result in errors up to 5mm[1] since it does not allow 3-dimensional positioning of maxilla acutely with respect to the basal skull. Especially, there were greater differences in anteroposterior and vertical changes.[13]This is likely due to the vertical dimension of maxillary position often being clinically determined intraoperatively. Recently, CAD/CAM technique or navigation assisted surgery have been applied to overcome the limitations of traditional surgical wafer[14,15] and various surgical methods using customized surgical guides or pre-bent plate have been introduced.[11,16]

Many previous studies have investigated the accuracy of CAD/CAM generated surgical wafers compared with manually fabricated surgical wafers. Kwon et al reported that the surgical accuracy of maxillary positioning with CAD/CAM wafer was comparable to conventional articulator generated wafer.[17] However, Song et al reported that the error of CAD/CAM wafer was less than 0.35mm, which was superior to the error of traditional wafer(0.94mm).[5] However, the maxillofacial surgeons have to consider some factors could create errors even in cases of using CAD/CAM technique: the error in processing 3D image, the error of 3D scan for a dental model, the errors in registration process and splint manufacturing, the difference of the condylar position.

The condylar position is also considered to be the most important factor affecting accuracy.[9] External reference landmark measurement is a must using conventional CAD/CAM wafer in order to control vertical positioning of maxilla, and a prudent intraoperative clinical assessment of maxillary vertical positioning by the surgeons is critical. The efficacy of external reference point was shown using a bone screw,[18] however this method can only decrease the vertical positioning error of anterior maxilla with limited control over vertical positioning error of posterior maxilla and maxillary plain angle.[10] in this regard, the greater errors can occur for inexperienced surgeons. Furthermore, in cases of large CO-CR discrepancy, the error in preoperative bite registration can lead to difficulty in delivering surgical plan accurately during operation with traditional surgical technique using mandibular autorotation. The intrinsic instability of mandible which the intermediate wafer is placed could interrupt the repositioning of maxilla in the desired position.

To solve these problems, customized surgical guides and navigation surgery have been developed. Locating guide and prebent titanium plated for orthognathic surgery using CAD/CAM technique and rapid prototype model was reported.[19] Another study reported the accuracy of customized bone cutting guide and a customized titanium plate.[16]

In this study, overlap errors using a threshold value smaller than 2mm were evaluated and the frequency of such error was used as a measurement of accuracy. The accuracy was 100% in 7 of 10 patients. Li et al described their experience in 6 patients using a CAD/CAM surgical guide consisted of osteotomy guide and repositioning guide. The results showed that the error in maxilla was within 1mm and the maximum error was 1.7mm.[11]

Marmulla and Muhling have reported that the median spatial malposition of the condyles with navigation was reduced to 0.7mm.[20] Nevertheless, navigation assisted surgery has an intrinsic limitation of the need to check many reference points during operation and due to high costs of commercial navigation systems, this system has not become a standard procedure.[16] Mandibular surgery first can overcome unstable CR position and inaccurate bite registration.[21] However, many patients in this study required a clockwise rotation of the maxillo-mandibular complex with maxillary posterior impaction, which is a difficult to achieve with mandibular surgery first. In this study, we have used the customized surgical guide consisted of occlusal based wafer, osteotomy, resection, and repositioning guides. The accuracy was compared with the conventional design CAD/CAM generated wafer. The average discrepancy [SD] of the maxilla was 0.78mm [SD] in conventional group and 0.77 mm [SD] in modified group, thus the results are considered acceptable in terms of accuracy. [9,22]

MSCT was used in the modified group for better 3-D anatomical detail of the maxillary anterior wall which is crucial in manufacturing an accurate modified surgical guide. This study included relatively small clinical cases and only measured the surface discrepancy between the bony surface with no vector consideration. Study with larger samples is required for validate the result of this study.

## Conclusion

Our study showed no significant differences between customized CAD/CAM surgical guide and occlusal based CAD/CAM wafer in terms of accuracy, thus the modified wafer does not provide better accuracy over conventional design. However, the modified water can be beneficial when an accurate maxillary repositioning is not reliable based on conventional wafer due to an unstable condylar position. Also, it can aid inexperienced surgeons to recognize the exact amount of bone reduction for maxillary repositioning.

## Supporting information

S1 File (XLSX)

## References

[1] Ellis E. Accuracy of model surgery: Evaluation of an old technique and introduction of a new one. J Oral Maxillofac Surg. 1990: 1161–1167 1698956 10.1016/0278-2391(90)90532-7

[2] Marchetti C, Bianchi A., Bassi M., Gori R., Lamberti C., Sarti A. <Mathematical modeling and numerical simulation in maxillo-facial virtual surgery (VISU). 2006.pdf>. J Craniofac Surg. 2006;17: 661–667 16877910 10.1097/00001665-200607000-00009

[3] Swennen GR, Mommaerts MY, Abeloos J, De Clercq C, Lamoral P, Neyt N, et al A cone-beam CT based technique to augment the 3D virtual skull model with a detailed dental surface. Int J Oral Maxillofac Surg. 2009;38: 48–57. 10.1016/j.ijom.2008.11.006 19118978

[4] Dai J, Tang M, Xin P, Hu G, Si J, Dong Y, et al Accurate movement of jaw segment in virtual 3D orthognathic surgery. J Craniofac Surg. 2014;25: e140–143. 10.1097/SCS.0000000000000414 24621754

[5] Song KG, Baek SH. Comparison of the accuracy of the three-dimensional virtual method and the conventional manual method for model surgery and intermediate wafer fabrication. Oral Surg Oral Med Oral Pathol Oral Radiol Endod. 2009;107: 13–21. 10.1016/j.tripleo.2008.06.002 18755612

[6] Cousley RR, Turner MJ. Digital model planning and computerized fabrication of orthognathic surgery wafers. J Orthod. 2014;41: 38–45. 10.1179/1465313313Y.0000000075 24235100

[7] Metzger MC, Hohlweg-Majert B, Schwarz U, Teschner M, Hammer B, Schmelzeisen R. Manufacturing splints for orthognathic surgery using a three-dimensional printer. Oral Surg Oral Med Oral Pathol Oral Radiol Endod. 2008;105: e1–7. 10.1016/j.tripleo.2007.07.040 18230371

[8] Aboul-Hosn Centenero S, Hernandez-Alfaro F. 3D planning in orthognathic surgery: CAD/CAM surgical splints and prediction of the soft and hard tissues results—our experience in 16 cases. J Craniomaxillofac Surg. 2012;40: 162–168. 10.1016/j.jcms.2011.03.014 21458285

[9] Schouman T, Rouch P, Imholz B, Fasel J, Courvoisier D, Scolozzi P. Accuracy evaluation of CAD/CAM generated splints in orthognathic surgery: a cadaveric study. Head Face Med. 2015;11: 24 10.1186/s13005-015-0082-9 26209339 PMC4514936

[10] Zinser MJ, Mischkowski RA, Sailer HF, Zoller JE. Computer-assisted orthognathic surgery: feasibility study using multiple CAD/CAM surgical splints. Oral Surg Oral Med Oral Pathol Oral Radiol. 2012;113: 673–687. 10.1016/j.oooo.2011.11.009 22668627

[11] Li B, Zhang L, Sun H, Yuan J, Shen SG, Wang X. A novel method of computer aided orthognathic surgery using individual CAD/CAM templates: a combination of osteotomy and repositioning guides. Br J Oral Maxillofac Surg. 2013;51: e239–244. 10.1016/j.bjoms.2013.03.007 23566536

[12] Jung H-D, Jung Y-S, Park JH, Park H-S. Recovery pattern of mandibular movement by active physical therapy after bilateral transoral vertical ramus osteotomy. Journal of oral and maxillofacial surgery. 2012;70: e431–e437 10.1016/j.joms.2012.02.033 22698299

[13] Zinser M, Zoeller J. Computer-Designed Splints for Surgical Transfer of 3D Orthognathic Planning. Facial Plast Surg. 2015;31: 474–490. 10.1055/s-0035-1565010 26579863

[14] Hernandez-Alfaro F, Guijarro-Martinez R. New protocol for three-dimensional surgical planning and CAD/CAM splint generation in orthognathic surgery: an in vitro and in vivo study. Int J Oral Maxillofac Surg. 2013;42: 1547–1556. 10.1016/j.ijom.2013.03.025 23768749

[15] Mazzoni S, Badiali G, Lancellotti L, Babbi L, Bianchi A, Marchetti C. Simulation-guided navigation: a new approach to improve intraoperative three-dimensional reproducibility during orthognathic surgery. J Craniofac Surg. 2010;21: 1698–1705. 10.1097/SCS.0b013e3181f3c6a8 21119403

[16] Mazzoni S, Bianchi A, Schiariti G, Badiali G, Marchetti C. Computer-aided design and computer-aided manufacturing cutting guides and customized titanium plates are useful in upper maxilla waferless repositioning. J Oral Maxillofac Surg. 2015;73: 701–707. 10.1016/j.joms.2014.10.028 25622881

[17] Kwon TG, Choi JW, Kyung HM, Park HS. Accuracy of maxillary repositioning in two-jaw surgery with conventional articulator model surgery versus virtual model surgery. Int J Oral Maxillofac Surg. 2014;43: 732–738. 10.1016/j.ijom.2013.11.009 24462125

[18] John W. Ferguson NHL. Control of vertical dimension during maxillary orthognathic surgery. A clinical trial comparing internal and external fixed reference points. J Craniomaxillofac Surg. 1992;20: 333–336 1464681 10.1016/s1010-5182(05)80360-9

[19] Bai S, Shang H, Liu Y, Zhao J, Zhao Y. Computer-aided design and computer-aided manufacturing locating guides accompanied with prebent titanium plates in orthognathic surgery. J Oral Maxillofac Surg. 2012;70: 2419–2426. 10.1016/j.joms.2011.12.017 22516840

[20] Marmulla R, Mühling J. Computer-Assisted Condyle Positioning in Orthognathic Surgery. Journal of Oral and Maxillofacial Surgery. 2007;65: 1963–1968. 10.1016/j.joms.2006.11.024 17884523

[21] Perez D, Ellis E, 3rd. Sequencing bimaxillary surgery: mandible first. 10.1016/j.joms.2010.10.053 21292376

[22] Hsu SS, Gateno J, Bell RB, Hirsch DL, Markiewicz MR, Teichgraeber JF, et al Accuracy of a computer-aided surgical simulation protocol for orthognathic surgery: a prospective multicenter study. J Oral Maxillofac Surg. 2013;71: 128–142. 10.1016/j.joms.2012.03.027 22695016 PMC3443525

